# New York Letter

**Published:** 1893-04

**Authors:** 


### NEW YORK LETTER.

NEW YORK, March 11th, 1893.

At a recent meeting of the County Medical Society, Dr. W. R. Coley read an interesting paper on the "Operative Treatment of Hernia in Children." He presented statistics showing that a very large percentage of hernia in children treated by mechanical methods are not cured, and that those who are cured the vast majority are well before the fifth or sixth year. He tabulated the following rules as indications for operating: 1. In cases of adherent omentum; 2. reducible hydrocele; 3. irreducible or strangulated hernia; 4. among the poorer classes, for they can not afford to carry out the mechanical treatment; 5. when mechanical treatment has been used a limited time, until the child is about five years of age, and failed. He also mentioned the following as grounds on which some are opposed to operating on children for hernia. 1. In children hernia are more amenable to mechanical treatment than in adults, but statistics show that if they are not healed within a limited time they are rarely cured. 2. They are not so apt to become strangulated in children as in adults. Though the danger is somewhat less in children it does exist and should not be made light of. 3. The operation for radical cure is more dangerous to life than the hernia supported by mechanical means. Statistics are not complete enough at present to settle this question.

While no single operation can be said to be *the* one for every case Dr. Coley prefers Bassini's method in most cases. He thinks many of the failures to cure in these operations are due to the material used in ligatures and sutures. Primary union is essential and if the sutures used be irritating a bad result is very apt to follow. In his operations Dr. Coley uses kangaroo tendon or ox peritoneum with which he has secured his most satisfactory results and he does not recommend the use of silk, but others have gained excellent results and had no symptoms of irritation from its use. Dr. De Garmo thought there was danger of compressing the cord in Bassini's operation and thus destroying the activity of the testicle but there can be little danger of this from the slight pressure that might be brought to bear upon the cord in this way, as Dr. Keyes states, that in operations for varicocele by the subcutaneous method he has frequently included the cord in the ligature without having atrophy of the testicle follow. [Syst. of Genito-Urinay Dis. Syph. and Dermat. Morrow p. 977].

Bassini's operation, which I think is not described in any general surgery, is very nicely depicted by Dr. Milliken in the *Now York Medical Record*, July 2, 1892. The most interesting points in this operation are the treatment of the sac and the method of closing the hernial track. The sac having been exposed and opened points of adhesion are tied off and if large amounts of omentum be present it is tied off preferably by the transfixion method. The sac is now separated from the cord and other adhesions that may exist well within the internal ring and uniform traction made at all points. Ligation by catgut is done by the transfixion method and the sac excised, leaving a pedicle about a quarter of an inch in length, which immediately retracts into the abdominal cavity. When congenital hernia exists the sac should be cut off one inch above the testis.

The cord being held out of the field the conjoined tendon is united to the edge of Poupart's ligament by a number of interrupted sutures, four to six sutures being sufficient. The edges of the divided external oblique tendon are now united by continous suture throughout its entire length. The skin wound is united by catgut sutures after the insertion of a small glass drainage tube about two to two and a half inches in length, into the lower angle of the wound, which can be removed at the end of thirty-six to forty-eight hours.

Prof. Paul F. Munde read a paper on "Unusual Cangenital Malformations of the Female Sexual Organs" reporting quite a number of most interesting cases that had come under his observation.

A young lady, single, had suffered a long time from dysmenorrhoea, which was relieved only by large doses of morphine. She had been treated by electricity, dilatation of the cervix, curetting, etc., but without result. One of the many physicians who treated her discovered that there was a double uterus and vagina one much smaller than the other, and it was from the smaller one that all her trouble arose. She came to Prof. Munde expecting to have her ovaries removed, as she had been told that she could never marry. This was refused and the division in the vagina was removed, the wall between the two uteri divided and the cavity packed with iodoform gauze. The patient made an uneventful recovery and when last heard from menstruated freely without pain.

A second case was that of a woman who was sent to the hospital supposed to be suffering from extra-uterine pregnancy. On examination she gave all the symptoms of this trouble and an operation was performed. On exposing the uterus there appeared to be foetation in the horn of the uterus. A sound was introduced and to the professors' surprise, passed about an inch deeper than it had on examination, but inclined away from the tumor while on examination it had passed rather towards the tumor. It was then withdrawn and introduced with the point inclined towards the tumor, when it passed as it had on the previous examination and to the same distance. This settled the question, the patient had a double uterus with a foetus in one side. She aborted the following day, and as one side of the uterus was much larger than the other it is supposed that the larger side held a child to which she had previously given birth. This case serves to add one more point to be differentiated in diagnosing ectopic gestation.

A third case was that of a young married lady who came to Dr. Munde' office to inquire why she did not bear children. On being asked if copulation was satisfactory she replied, though hesitatingly, that it was. On examination there was found a vagina little more than half an inch deep, no uterus, and ovaries about the size of half a pea. She, of course, had never menstruated. All these cases, so far as external appearances were concerned, including the appearance of the external genitals, were perfectly formed women, with nothing to cause one to suspect an abnormal development of any part, and in two of them the malformation was exposed only after the most thorough examination.

At a recent meeting of the Academy of Medicine Prof. Beverly Robinson read a paper on the "Diagnosis and Treatment of Pleurisy" in which he presented many most interesting points. He mentioned many instances in which infection had followed aspirating in case of pleurisy and advised the withdrawal of fluid from the pleural cavity whenever it was found but thought it best not to withdraw it all at one time if the amount were large. In aspirating it is necessary to use a good sized needle, and one in which you are sure of the temper for three cases were mentioned in which small needles were used with the result of their being broken off and lost in the cavity. Two of the cases died and the needles were found resting harmlessly at the bottom of the pleural cavity the other case recovered and the needle was never heard from, but nevertheless no one would feel any the more comfortable if he knew that a needle was resting so near his heart.

Dr. Janeway thought the most difficult cases to diagnose were those in which there was a saculated empyema in the upper portion of the chest when the symptoms will closely resemble pneumonia. He had seen two cases of this character one of which was diagnosed at the autopsy and the other followed so quickly that he was on his guard for it. He gave some interesting points in the differential diagnosis of pleurisy and his standing as a diagnostician make them most valuable. It has been said of Dr. Janeway that his success as a diagnostician is the result of his close observation of the most minute details. In pneumonia the chest wall is not enlarged and the heart is not displaced while in pleurisy with effusion or empyema both are present. In pleurisy with effusion whispering broncophony is present while in empyema it is not. In many cases it is not possible to say from the general condition of the patient what the character of the fluid is, as was evidenced in the case of a noted physician last spring. The patient was suffering from septicæmia and developed pneumonia, later signs of fluid in the pleural cavity appeared. It was natural to expect to find pus in such a case, but on aspirating clear serum was found.

Another condition that may complicate the diagnosis of pleurisy with effusion is that of an aneurism pressing on the bronchial tube and obliterating it, the lobe of the lung with which it communicates becoming filled with mucus. Such cases are only diagnosed by other symptoms of aneurism being present. Again, there may be enlargement of the liver or spleen, held in position by adhesions below so that it extends upward, compressing the lung and even displacing the heart. There may be fluid in the pleural cavity with symptoms that lead us to suspect pus but which proves to be serum on aspiration, in such cases it is not rare to find an abscess of the liver or spleen.

Dr. Loomis spoke of the importance of pleurisy at the apex of the lungs. Here where, from recent histological researches, the lymphatics from the bronchial glands are found to open is a dangerous point for pleurisy to develop, for, if our supposition that the tubercle bacilli enter through these glands, is correct, here the least symptom of inflammation should lead us to suspect the development of tuberculosis and to treat our patients accordingly.

